# Adipocyte pyroptosis occurs in omental tumor microenvironment and is associated with chemoresistance of ovarian cancer

**DOI:** 10.1186/s12929-024-01051-4

**Published:** 2024-06-11

**Authors:** Chang-Ni Lin, Yu-Ling Liang, Hsing-Fen Tsai, Pei-Ying Wu, Lan-Yin Huang, Yu-Han Lin, Chieh-Yi Kang, Chao-Ling Yao, Meng-Ru Shen, Keng-Fu Hsu

**Affiliations:** 1 Department of Obstetrics and Gynecology, College of Medicine, National Cheng Kung University Hospital, National Cheng Kung University, 138, Sheng-Li Road, Tainan, 70428 Taiwan; 2 Department of Obstetrics and Gynecology, Chi-Mei Medical Center, Tainan, Taiwan; 3 Department of Chemical Engineering, National Cheng Kung University, Tainan, Taiwan; 4 Institute of Clinical Medicine, College of Medicine, National Cheng Kung University, Tainan, Taiwan

**Keywords:** Ovarian cancer, Omentum metastasis, Inflammation, Adipocyte pyroptosis, Chemoresistance

## Abstract

**Background:**

Ovarian carcinoma (OC) is a fatal malignancy, with most patients experiencing recurrence and resistance to chemotherapy. In contrast to hematogenous metastasizing tumors, ovarian cancer cells disseminate within the peritoneal cavity, especially the omentum. Previously, we reported omental crown-like structure (CLS) number is associated with poor prognosis of advanced-stage OC. CLS that have pathologic features of a dead or dying adipocyte was surrounded by several macrophages is well known a histologic hallmark for inflammatory adipose tissue. In this study, we attempted to clarify the interaction between metastatic ovarian cancer cells and omental CLS, and to formulate a therapeutic strategy for advanced-stage ovarian cancer.

**Methods:**

A three-cell (including OC cells, adipocytes and macrophages) coculture model was established to mimic the omental tumor microenvironment (TME) of ovarian cancer. Caspase-1 activity, ATP and free fatty acids (FFA) levels were detected by commercial kits. An adipocyte organoid model was established to assess macrophages migration and infiltration. In vitro and in vivo experiments were performed for functional assays and therapeutic effect evaluations. Clinical OC tissue samples were collected for immunochemistry stain and statistics analysis.

**Results:**

In three-cell coculture model, OC cells-derived IL-6 and IL-8 could induce the occurrence of pyroptosis in omental adipocytes. The pyroptotic adipocytes release ATP to increase macrophage infiltration, release FFA into TME, uptake by OC cells to increase chemoresistance. From OC tumor samples study, we demonstrated patients with high gasdermin D (GSDMD) expression in omental adipocytes is highly correlated with chemoresistance and poor outcome in advanced-stage OC. In animal model, by pyroptosis inhibitor, DSF, effectively retarded tumor growth and prolonged mice survival.

**Conclusions:**

Omental adipocyte pyroptosis may contribute the chemoresistance in advanced stage OC. Omental adipocytes could release FFA and ATP through the GSDMD-mediate pyroptosis to induce chemoresistance and macrophages infiltration resulting the poor prognosis in advanced-stage OC. Inhibition of adipocyte pyroptosis may be a potential therapeutic modality in advanced-stage OC with omentum metastasis.

**Supplementary Information:**

The online version contains supplementary material available at 10.1186/s12929-024-01051-4.

## Introduction

Ovarian carcinoma (OC) is the gynecologic malignancy with the highest case-to-fatality ratio, with most patients experiencing recurrent disease that is resistant to chemotherapy [1]. In contrast to tumors that undergo hematogenous metastasis, OC primarily disseminates within the peritoneal cavity [2], especially the omentum. The omentum, a lipid-rich tissue, has been reported to release fatty acids from adipocytes, which are used by adjacent cancer cells to fuel their growth, proliferation and increase chemoresistance [3, 4]. However, the mechanisms between cells in the omentum and tumor cells with chemotherapy resistance remain largely unknown.

Once OC occur metastasis, a complex tumor microenvironment (TME) in omentum has been created including metastatic OC cells, adipocytes, immune cells, etc. Previously, we reported that advanced-stage high grade serous ovarian carcinoma (HGSOC) patients with omental crown-like structure (CLS) present had a poorer prognosis than those without CLS present [5]. Dead or dying adipocytes are surrounded by macrophages to form the characteristic CLS, which has been characterized as a histologic hallmark of adipose tissue with inflammation. More importantly, dying adipocytes may release some molecules, such as ATP, that are known to recruit macrophages [6]. So far, how omental adipocyte develop death signal and how macrophages infiltrate into omentum, especial around adipocyte, were still not clear.

Recently, an inflammatory programmed cell death characterized by gasdermin-mediated cell death, pyroptosis, was described [7, 8]. There are four main distinct signaling pathways that have been identified to induce pyroptosis including the canonical and noncanonical inflammasome pathways, apoptotic caspase-mediated pathway, and granzyme-based pathway [8]. In these pathways, gasdermin proteins are the final executioners, and they need to be cleaved by upstream caspases or granzymes to promote pore formation in cell membrane, and then release of intracellular contents, such as IL-1β, IL-18 and ATP [9, 10]. Accumulating evidences indicate that pyroptosis can affect the development of tumors, and inflammasomes especially and may serve as positive or negative regulators of tumorigenesis [11, 12]. In this study, based on our previous observation of omental CLS formation in OC, we aimed to explore the initiation of adipocyte pyroptosis in the omental TME, molecular regulation between adipocytes and OC cells, and try to find a therapeutic strategy in advanced-stage OC.

## Materials and methods

### Cell culture and an adipocyte organoid model

Human ovarian cancer cell lines (SKOV3 and OVCAR4), human monocytic cell lines (THP-1 and U937) were cultured in RPMI 1640 medium (Thermo Fisher Scientific, Waltham, MA, USA) containing 10% fetal bovine serum (HyClone, GE Healthcare Life Sciences, South Logan, Utah, USA). The seeding number of cells was listed in Additional file 1: Fig. S1A. A mouse cells 3T3L1, were differentiated to adipocyte-like cells in RPMI with insulin (10 µg/ml), dexamethasone (1 µM) and 1-methyl-3-isobutyl-xanthine (0.5 mM) from Day1-3 and with insulin (10 µg/ml) from day 4–7, then maintained in RPMI with insulin (5 µg/ml) as showed in Additional file 1: Fig. S1B. Immortalized normal uterine cervical epithelial cells (iNECs) were developed as described in previous reports [13–15]. In brief, primary normal cervical epithelial cells were isolated from women who underwent hysterectomy for benign uterine diseases. The cells were further transduced with an amphotropic LXSN retroviral vector expressing HPV type 16 E6 and E7 and serially passaged. All cells were incubated in a humidified atmosphere at 37 °C with 5% CO_2_.

For human adipocyte isolation, approximately 5 × 5 cm^2^ omentum specimens were collected from each five female patients with benign gynecologic disease treated at the National Cheng Kung University Hospital. Informed consent was obtained from each subject before surgery, and the study was approved by the Institutional Review Board at the National Cheng Kung University Hospital (IRB number B-ER-110-484). The omentum was collected and adipocyte isolation was performed within 4 h as previous report [16] with some modifications. The omental tissue was first cut into small pieces, with collagenase digestion, fractionation and centrifugation. The adipose tissue had separated into four layers: (a) a clear yellow layer that contained lipids released from dead adipocytes, (b) an opaque white‒yellow layer that contained mature adipocytes, (c) a clear brownish liquid that contained the enzyme solution, and (d) a red pellet that contained the stromal vascular fraction. Mature omental adipocytes (OA) (approximately 1 × 10^7^ to 2 × 10^7^ cells could be harvested) from b layer were cocultured with SKOV3, THP-1 or iNEC cells for future experiments.

3T3L1-GPF (3T3L1 adipocytes cell with GFP transfection) cells were cultured in RPMI 1640 medium with 0.25% cellulose (M0262; Sigma‒Aldrich^®^) for 4 days. Numerous suspended organoid spheres were clearly visible under a microscope (Additional file 1: Fig. S3A). These spheres were harvested by centrifugation for 5 min at 1000 r.p.m. and replated with THP-1-RFP (THP-1 macrophages cell with RFP transfection) cells in conditioned medium from a 3T3L1/THP-1/SKOV3 coculture to mimic tumor environment conditions. At the same time, 3T3L1-GPF spheres with THP-1-RFP cells in conditioned medium from 3T3L1/THP-1/iNEC were used as a control to mimic non-tumor environment conditions. For calculation of the infiltrating macrophages (THP-1-RFP cells) in the adipocytes sphere (3T3L1-GFP cells), serial section of the adipocyte sphere at 1 μm at Z stack was performed and number of THP-1-RFP cell was calculated. For demonstrating the migration of THP-1-RFP cells in adipocyte organoid model, video was done by collecting the serial time-point images obtained by an ImageXpress^Micro^ wide-field fluorescence microscope (Delta-vision, Molecular Devices, USA) every 5 min for two hours, every hour for 3 h, and then every 2 h for further 24 h.

### Cell viability assay

Adipocyte cell viability was assessed by calcein AM staining, and apoptosis assessed with PI /Annexin V staining after coculture, then quantified by flow cytometry analysis. The mean fluorescence intensity (MFI) was determined with FCSalyzer 0–5 software. The data are expressed as the mean values ± standard errors of the means of three experiments. Additionally, the colorimetric MTT assay was used to assess tumor cell viability.

### Western blot analysis

Thirty micrograms of protein was subjected to 10% SDS‒PAGE and transferred to polyvinylidene fluoride (PVDF) membranes (IPVH00010; Millipore). The primary antibodies (Abs) were anti-NLRP3 (ab23094-1; Abcam), anti-caspase-1 (ALX-210-804-C100; Enzo Life Sciences, Inc.), anti-GSDMD (AF4012; Affinity Biosciences), anti-acetyl-STAT3 (#2523; Cell Signaling, Technology, Inc.), anti-STAT3 (#9132; Cell Signaling, Technology, Inc.), anti-ACSL4 (sc-365,230; Santa Cruz Biotechnology, Inc.) and anti-CPT1B (#2217-1-AP; Proteintech Group, Inc.). The secondary antibodies were anti-rabbit and anti-mouse HRP conjugates (1:10,000 dilution; A0545 and A9044; Sigma‒Aldrich). The blocking/dilution reagent was 5% skim milk in Tris-buffered saline containing 0.05% Tween 20 (TBST). Proteins were visualized using X-ray film and an enhanced chemiluminescence system (Millipore). Protein expression levels were determined by using ImageJ software.

### Confocal imaging and immunocytochemistry

Since recombinant human IL-6 and IL-8 had biologic effect on 3T3L1 adipocytes [17], differentiated 3T3L1 adipocytes were cultured in chamber slides with/without recombinant human IL-6 (206-IL; R&D Systems) and IL-8 (208-IL; R&D Systems) treatment for 3 days. The cells were fixed in 4% paraformaldehyde for 15 min at room temperature (RT). They were washed with ice-cold phosphate-buffered saline (PBS) and then incubated for 10 min in 0.25% Triton X-100 in PBS. Bovine serum albumin (BSA; 1%) in PBS with Tween 20 (PBST) was then added to the cells for 30 min for blocking, and then anti-GSDMD antibody (AF4012; Affinit Bioscience) was added and incubated overnight at 4 °C. After washing with PBST, the samples were treated with a PE-conjugated phalloidin (sc-363,795; Santa Cruz Biotechnology) and Alexa488-conjugated secondary antibody (5230 − 0385; SeraCare Life Sciences Inc.) in PBS for 1 h at RT in the dark. The immunofluorescence staining results were observed under a confocal-based fluorescence microscope (Delta-vision, Molecular Devices, USA). The GSDMD dots were defined as GSDMD stain on the phalloidin for 50 pixels of a cell outermost, and these puncta were calculated by MetaXpress software, and the number of DAPI stain was as the cell number per view. Finally, the puncta/DAPI is defined as GSDMD dot number per cell.

### Cytokine array

A human cytokine antibody array kit (ab133997) was used to detect cytokines in condition medium according to the manufacturer’s instructions. Capture antibodies were supplied spotted on a membrane. Each pair of spots represented a different analyte, the map was showed on Additional file 1: Fig. S2A. The CM were added (1 ml of CM sample to each membrane) and incubation for overnight at 4℃. After remove CM and wash membrane with wash buffer, then added paired biotinylated detector antibodies and streptavidin HRP. The cytokine array were analyzed and visualized using X-ray film and chemiluminescence system after remove streptavidin HRP and wash with wash buffer.

### Quantitative RT‒PCR analysis

A NucleoSpin RNA extraction kit (2104/006; MACHEREY-GNAGEL) was used to extract total RNA according to the manufacturer’s instructions. cDNAs were synthesized using the ImPromII^™^ Reverse Transcription System (A3800; Promega). PCR was carried out on a StepOnePlus Real Time PCR System (Applied Biosystems). The reaction parameters were 10 min at 95°C, followed by 45 cycles of 15 s at 95°C and 60 s at 60°C. The melt curve conditions were set at 95°C for 15 s, 60°C for 60 s and 95°C for 15 s. Triplicate mean values were calculated using GAPDH gene transcription as the reference for normalization. The sequences of the primers used for qPCR analysis were: IL-6, 5’-AAAGAGGCACTGGCAGAAAA-3’ (forward) and 5’-TTTCACCAGGCAAGTCTCCT-3’ (reverse); IL-8, 5’-GTGCAGTTTTGCCAAGGAGT-3’ (forward) and 5’-CTCTGCACCCAGTTTTCCTT-3’ (reverse); and CCL5, 5’-ATCTGCCTCCCCATATTCCT-3’ (forward) and 5’-GCACTTGCCACTGGTGTAGA-3’ (reverse).

### ELISA

The IL-6 and IL-8 cytokine levels in OC patients’ ascites were measured by sandwich enzyme-linked immunosorbent assay (ELISA). Briefly, the capture and detection antibodies, standard, and streptavidin-conjugated HRP were provided by the ELISA kit manufacturer (R&D Systems). The TMB Microwell Peroxidase Substrate System (Clinical Science Products, Inc.) and an absorbance microplate reader (Sunrise™; TECAN) were used for colorimetric detection and quantitation.

### Caspase-1 activity assay

Caspase-1 activity measurement was by a commercial kit (ab39412; Abcam) which is based on the cleavage of the substrate YVAD-AFC (AFC: 7-amino-4-trifluoromethyl coumarin) and quantification of free AFC (Ex/Em = 400/505 nm) using a fluorescence microtiter plate reader (Fluoroskan Ascent, Thermo).

### Cell migration/invasion assay

Transwell chambers with polycarbonate membrane filters (8-µm pore size; Corning Life Sciences) were coated with 50 µl Matrigel solution (5X dilution in RPMI 1640 medium; BD Biosciences). SKOV3 cells (2 × 10^4^) were added to the upper chamber. The lower chamber was filled with 3-cell coculture conditioned medium with/without 100 µM SSO. RPMI medium supplemented with 10% FBS was used as the negative control. After 24 h of incubation at 37 °C, the upper surface of the filter was washed with PBS, and nonmigratory cells were removed with a cotton swab. The remaining cells on the lower surface of the filter were fixed with cold methanol and stained with a Giemsa solution (Merck). Invasive cells were quantified under a microscope at 200X magnification.

### Lipid staining and free fatty acid quantitation

Free fatty acid detection was based on the conversion of long-chain fatty acids to their CoA derivatives, which are subsequently oxidized with the concomitant generation of a fluorescence signal (Ex/Em = 535/587 nm). Fluorescence levels were detected using a fluorescence microtiter plate reader (Fluoroskan Ascent, Thermo). The fluorometric detection protocol used a commercially available kit (ab65341; Abcam) according to the manufacturer’s instructions. The Lipid (Oil Red O) Staining Kit is suitable for selective staining and detection of neutral lipids in cultured cell.

### Adenosine triphosphate (ATP) detection

Extracellular ATP was detected using bioluminescence to measure the level of ATP that was released into the extracellular environment as a result of cell death, stress or activation. In this study, we measured extracellular APT levels in 3-cell coculture CM and OC patients’s ascites. The ATP concentration detected by a microplate luminometer (Luminoskan Ascent, Thermo) with a commercially available kit (GA5010; Promega) according to the manufacturer’s instructions.

### Transmission Electron Microscopy (TEM)

3T3L1 cells after coculture with THP-1/iNEC or THP-1/SKOV3 for 7 days were fixed in 2.5% glutaraldehyde in PBS overnight at 4 °C and postfixed in 1% osmium tetroxide (OsO4) at room temperature for 1 h. The cells were then dehydrated through an ascending grade series of ethanol at RT (25% for 10 min, 50%, 75%, 95%, 100% at 20 min each) and then 100% acetone for 20 min twice. The cells were then embedded in fresh resin and polymerized at 60 °C for at least 24 h. Ultra-thin sections of the cells (80–100 nm) were then cut from the blocks and then stained with uranyl acetate. The cells were viewed with a JEOL 1200 transmission microscope at 80 kV (JEOL, Tokyo, Japan).

### Immunohistochemistry and immunofluorescence staining

Paraffin-embedded omentum tissue samples from advanced stage OC patients were immunostained with an anti-CD36 antibody (18836-1-AP; Proteintech) and anti-CD68 antibody (M0814; Dako) for immunohistochemistry; anti-perilipin antibody (sc-390,169, Santa Cruz Biotechnology, Inc.) and anti-GSDMD antibody (1:100; AF4012; Affinity Biosciences) for immunofluorescence. Then, a horseradish peroxidase (HRP)-conjugated immunoglobulin G (IgG) antibody was added and incubated for 1 h, and the specimens were analyzed by using avidin and biotinylated enzyme complex (ABC) detection; or added fluorophore-conjugated IgG for imaging by fluorescence microscopy. Immunostaining images were acquired under a ZEISS Axio Imager D2 microscope, and quantification was performed using Nuance imaging software (version 3.0.2, PerkinElmer. GSDMD score were developed from positive GSDMD stain (yellow color) area in the area of three 200× fields (% of area). By the median value of GSDMD score as cut point, OC patients were divided into the GSDMD high expression and GSDMD low expression groups, and patient prognosis was analyzed according the GSDMD expression.

### Xenograft mouse model

Seven-week-old NOD/SCID mice were obtained from the Laboratory Animal Center, College of Medicine, National Cheng Kung University, Taiwan. The housing and experimental animal procedures were approved by the Institutional Animal Care and Use Committee of NCKU (approval number IACUC109133). SKOV3 cells transfected with GFP/Luciferase (2 × 10^6^/100 µL PBS) were intraperitoneally injected into twenty mice. Treatment started two weeks later after tumor inoculation. The mice were divided into 4 groups: Group A mice received no treatment, Group B mice was intraperitoneally treated with cisplatin (4 mg/kg) twice per week, Group C mice received DSF (50 mg/kg) treatment twice per week and Group D mice received cisplatin (4 mg/kg) combined with DSF (50 mg/kg) treatment twice per week. At the 8th weeks after tumor injection, all mice were sacrificed for preventing excessive tumor burden by the regulation of the animal care.

### Patient enrollment

Patients who underwent surgery for ovarian cancer in National Cheng Kung University Hospital (NCKUH), with final pathologic report as the 2014 International Federation of Gynecology and Obstetrics (FIGO) stage III/IV disease, high grade serous ovarian cancer were consecutively enrolled in this study. A total of 163 stage III/IV advanced-stage OC patients were diagnosed between 2015 and 2021 at National Cheng Kung University Hospital (NCKUH). Protocol was approved by Institutional Review Board of NCKUH (IRB number B-ER-110-484). Written informed consent for participation in the study was obtained from the participants. Patient omental tissue samples, ascites and clinical information were collected. Survival time was calculated from the date of surgery. Progression free survival was determined based on the date of first progression or death. All patients received adjuvant platinum-based chemotherapy except the patients whose performance status was too poor to receive it. Patients with disease progression or disease recurrence < 6 months after discontinuing chemotherapy were defined as chemoresistant, whereas patients without a recurrence or with recurrence ≥ 6 months after discontinuing chemotherapy were defined as chemosensitive.

### Statistical analysis

Student’s t test (two-tailed), correlation and survival analyses were performed using GraphPad Prism 8 software to identify significant differences. Statistical significance was set at *p* < 0.05. Data are expressed as the mean ± standard error of the mean (SEM) based on three independent experiments.

## Results

### Omental adipocytes and 3T3L1 adipocytes undergo pyroptosis rather than apoptosis in 3-cell coculture system or omental TME of OC

**Fig. 1:**
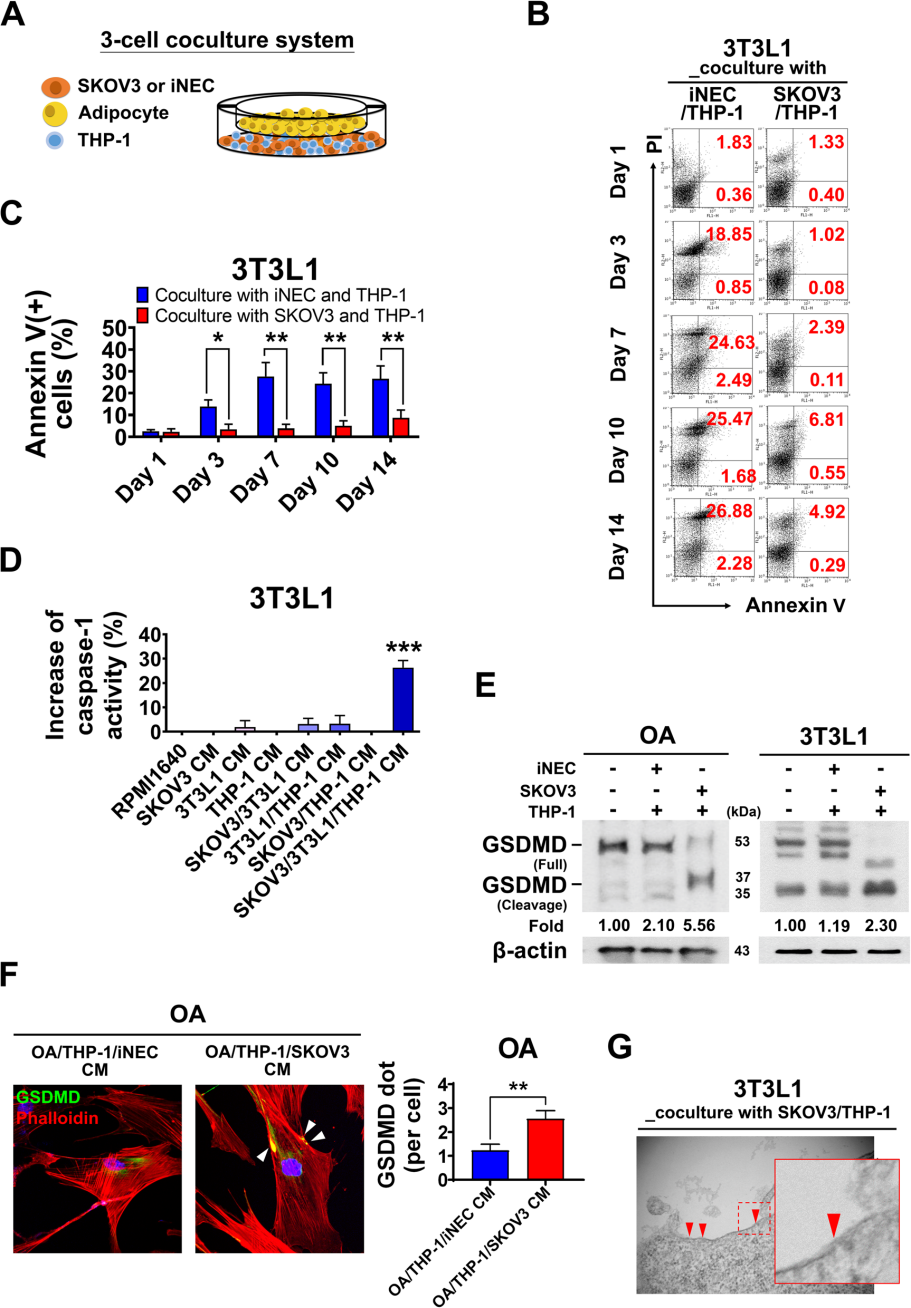
Pyroptotic cell death occurrence in adipocytes was observed in the 3-cell coculture system. **A** A cartoon diagram shows the 3- cell coculture system, including ovarian cancer cell (SKOV3) or normal epithelial cell (iNEC), adipocyte (primary culture omental adipocyte, OA or differentiated 3T3L1 adipocyte cells (3T3L1) and THP-1 macrophage (THP-1), which mimic the omental tumor microenvironment. **B**,** C** When cocultured with ovarian cancer SKOV3, the 3T3L1 adipocytes developed less apoptosis, compared to coculture with normal epithelial cell iNEC. By PI, Annexin V staining, the percentage of apoptotic 3T3L1 adipocytes increased to 27.12% when coculture with iNEC/THP-1 cells, while only 2.50% when coculture with SKOV3/THP-1 cells at Day 7. The quantification results in different days were shown in (**C**). **D** The increase of caspase-1 activity of 3T3L1 adipocytes was significantly higher when 3T3L1 adipocytes treated with SKOV3/3T3L1/THP-1 3-cell coculture condition medium (CM) than those with other condition medium. **E** The expression level of cleaved-GSDMD in adipocytes was obviously increased when cocultured with SKOV3. The expression of cleaved-GSDMD in OA, 3T3L1 adipocytes was 5.56-fold and 2.30-fold when cocultured with THP-1/SKOV3 cells than cultured alone; the expression of cleaved-GSDMD in OA, 3T3L1 adipocytes was 2.1-fold in OA and 1.19-fold in 3T3L1 adipocytes when coculture with THP-1/iNEC cells. The molecular weight of cleaved-GSDMD in OA cells from human is 37 kDa, in 3T3L1 adipocytes cells from mice is 35 kDa. **F** By GSDMD (in green) and Phalloidin (in red) immunofluorescence double staining in OA, the confocal image analysis showed more GSDMD yellow dots (arrow head) on the cell membrane of OA when treated with OA/THP-1/SKOV3 CM compared with OA/THP-1 /iNEC CM, (GSDMD dots per cell, mean: 2.55 vs. 1.23, *p* < 0.01 ). **G** Transmission electron microscopy (TEM) images showed some GSDMD-formed pores (arrow head, pore diameter less than 30 μm) in the 3T3L1 adipocyte cell membrane after coculture with SKOV3/THP-1 cells. ∗*p* < 0.05; ∗∗*p* < 0.01; ∗∗∗*p* < 0.001

To explore the possible interplay among ovarian cancer cells, adipocytes and macrophages in the omental TME of OC, a 3-cell coculture system was established as shown in Fig. 1A. The indicated cell for analysis was plated in the upper chamber, while the other 2 type cells were plated in the lower chamber. The seeding number of cells was listed in Additional file 1: Fig. S1A, and the protocol of 3T3L1 adipocytes differentiation was described in Additional file 1: Fig. S1B. Because leukemia monocyte cell line THP-1 could be differentiated to macrophage in coculture with tumor cells, we used THP-1 in 3-cell coculture. In the 3-cell coculture system, we found about 60% of THP-1 cells develop to CD68 positive macrophages after cocultured with 3T3L1/iNEC or 3T3L1/SKOV3 for 3 days (Additional file 1: Fig. S1C).

To understand the viability and apoptosis of adipocytes in the omental TME in OC, by the 3-cell coculture system, we performed viability assay with calcein AM staining and Annexin V/PI double stain of 3T3L1 adipocytes cells after coculture with OC cell or normal epithelium cell (iNEC) for 1, 3, 7, 10 or 14 days. The number of viable 3T3L1 adipocytes cells (calcein AM (+) cells) decreases with coculture days (Additional file 1: Fig. S1D). However, the percentage of viable 3T3L1 adipocytes cells cocultured with SKOV3/THP-1 was higher than it cocultured with iNEC/THP-1, especially at the day 7. Interestingly, high percentage of Annexin V positive cells occurred in 3T3L1 cocultured with iNEC/THP-1 as compared with it cocultured with SKOV3/THP-1, especially at the day 7 (Fig. 1B, C). The percentage of annexin V positive cells, i.e. apoptotic cell, of 3T3L1 adipocytes cocultured with SKOV3/THP-1 was constant low during culture period. Similar results were also observed in 3T3L1 adipocytes after coculture with another ovarian cancer cell OVCAR4 and U937 macrophages cell (Additional file 1: Fig. S1E-G). These results suggested that apoptotic cell death of adipocytes rarely occur in 3-cell coculture system or omental TME in OC.

Because omentum, in addition of adipose tissue, is known with function of immune-regulation [18], we next explored whether inflammation-related cell death, namely pyroptosis, occurs in adipocytes in the omental TME. In pyroptotic cells, caspase-1 is activated by inflammasomes and then cleaves gasdermin D (GSDMD) to promote membrane pore formation during programmed cell death. Pyroptosis can be evaluated by quantifying intracellular caspase-1 activation and GSDMD cleavage. By treatment of 3T3L1 adipocytes with 3T3L1/THP-1/SKOV3 CM up to 7 days, we observed the NLRP3 expression and cleavage-form GSDMD in 3T3L1 adipocytes cells obviously increased at day 3 (Additional file 1: Fig. S1H). In this 3-cell coculture system, the cleavage-form GSDMD of THP-1 macrophages, SKOV3 did not increase (Additional file 1: Fig. S1I). The caspase-1 activity significantly increased by 30% in 3T3L1 adipocytes cells after treatment with 3T3L1/THP-1/SKOV3 CM for 3 days (Fig. 1D) compared with other CM treatments. The caspase-1 activity of 3T3L1 adipocytes also significantly increased when treated with another OC, OVCAR4/3T3L1/THP-1 CM, or another U397 macrophages i.e. SKOV3/3T3L1//U397 CM (Additional file 1: Fig. S1J-L). The cleavage GSDMD expression increased about 5.56-fold and 2.30-fold in human omental adipocytes (OA) and 3T3L1 adipocytes cells when cocultured with THP-1/SKOV3 cells for 7 days (Fig. 1E). While increased about 1.36-fold and 1.86-fold in OA and 3T3L1 adipocytes when cocultured with THP-1/SKOV3 cells for 3 days (Additional file 1: Fig. S1M).

Furthermore, by immunofluorescence double staining of GSDMD and phalloidin, we observed more yellow GSDMD dots (arrow head) in cell membrane of OA when treated with OA/THP-1/SKOV3 CM than with OA/THP-1 /iNEC CM, (GSDMD dots per cell, mean: 2.55 vs. 1.23, *p* < 0.01 ) (Fig. 1F). By transmission electron microscopy (TEM), we also observed some pore formation (less than 30 μm) in the cell membrane of 3T3L1 adipocytes when coculture with THP-1/SKOV3 for 7 days (Fig. 1G). In the meantime, 3T3L1 adipocytes exhibited characteristics of apoptosis after coculture with THP-1/iNEC for 7 days (Additional file 1: Fig. S1N). All these data supports that adipocytes in 3-cell culture system occur pyroptosis rather than apoptosis.

### OC-derived IL-6 and IL-8 induce adipocyte pyroptosis in the in 3-cell coculture system or omental TME of OC

**Fig. 2:**
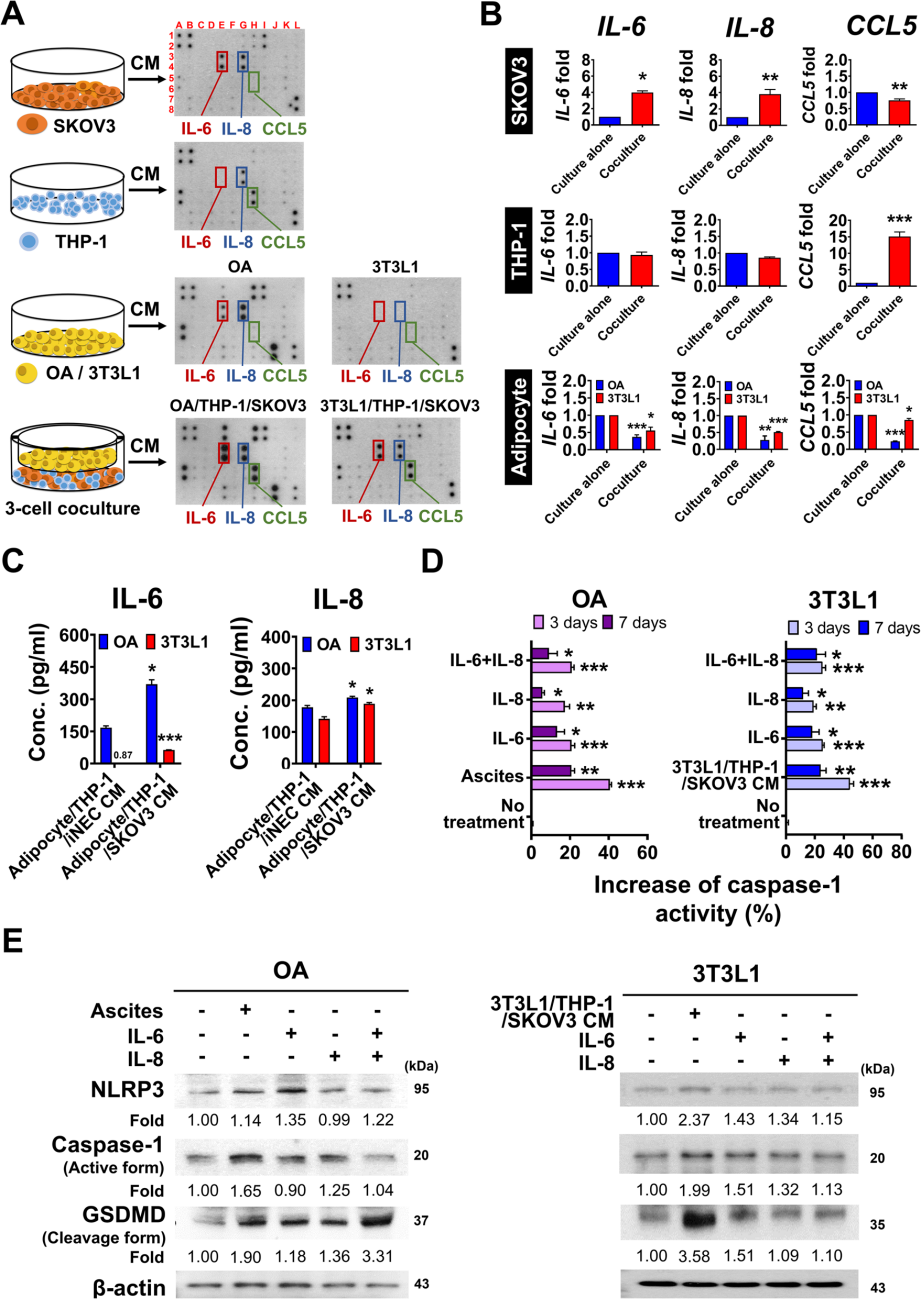
IL-6, IL-8 were abundantly in 3-cell coculture system and associated with adipocyte pyroptosis. **A** By cytokine arrays analysis, the IL-6, IL-8 and CCL5 levels in mediums from OA/THP-1/SKOV3, 3T3L1/THP-1/SKOV3 coculture were increased. **B** Expression of IL-6, IL-8 and CCL5 by quantitative PCR in SKOV3, THP-1 and OA, 3T3L1 adipocyte cells in different culture condition. The results showed an increase in the IL-6 expression level of 3.99-fold, IL-8 expression level of 3.81-fold in SKOV3 cells, and the CCL5 expression level of 15.07-fold in THP-1 cells in 3-cell coculture than cells in culture alone. The expression levels of IL-6, IL-8 and CCL5 were all reduced in OA, 3T3L1 adipocyte after 3-cell coculture than cells in culture alone. **C** In ELISA analysis, the concentrations of IL-6 (mean: 370.36 pg/ml) and IL-8 (mean: 208.93 pg/ml) in OA/THP-1/SKOV3 CM were significantly higher than that in OA/THP-1/iNEC CM. Also, the levels of IL-6 (mean: 63 pg/ml) and IL-8 (mean: 189 pg/ml) in 3T3L1/THP-1/SKOV3 CM were significantly higher than that in 3T3L1/THP-1/iNEC CM. **D** Both OA, 3T3L1 adipocyte cells increased caspase-1 activity when treated with IL-6, IL-8, ovarian cancer patient ascites or 3T3L1/THP-1/SKOV3 CM at 3, 7 days compared to no treatment. After 3 days treatment, in OA, caspase-1 activity increased 40.63% after treated with ascites from ovarian cancer patient, increased 20.78% after IL-6 (370 pg/ml) treatment, increased 17.37% after IL-8 (209 pg/ml) treatment and increased 20.90% after combined IL-6 and IL-8 treatment. In 3T3L1 adipocytes, the caspase-1 activity increased 44.31% after 3T3L1/THP-1/SKOV3 CM treatment, increased 25.48% after IL-6 (63 pg/ml) treatment, increased 19.17% after IL-8 (189 pg/ml) treatment and increased 25.2% after combined IL-6 and IL-8 treatment. **E** The NLRP3/caspase-1/GSDMD pathway was activated by increased cleavage -form of GSDMD in OA and 3T3L1 adipocytes after 3 day treatment with ascites, 3T3L1/THP-1/SKOV3 CM, IL-6, IL-8 or combined IL-6 and IL-8 treatment. The OA treated with OC patient’s ascites, 3T3L1 adipocytes treated with 3T3L1/THP-1/SKOV3 CM, respectively were as a positive control. ∗*p* < 0.05; ∗∗*p* < 0.01; ∗∗∗*p* < 0.001

Pyroptosis signaling pathways is known to classify into canonical and noncanonical pathways. However, both pyroptotic pathways require external stimuli, such as hypoxia, injury, toxins, pathogens or chemotherapeutic drugs, to induce intracellular signal transduction [7]. In the TME, cytokines play a crucial role of biological processes of cells including proliferation, differentiation, and migration. Therefore, adipocyte pyroptosis may be regulated by cytokines in omental TME. We next screen potential cytokines in CM that could change by cytokine array assay (Additional file 1: Fig. S2A). Compared with the levels in the CM of parental cells, the levels of IL-6, IL-8 and CCL5 in the 3-cell coculture CM were obviously increased (Fig. 2A) (Additional file 1: Fig. S2B). We further used qPCR to validate the transcription level of *IL-6*, *IL-8* and *CCL5* in individual cell to find where they came from. We found that the *IL-6*, *IL-8* transcriptional level was increased by 3.99-fold, 3.81-fold respectively of SKOV3 cells in 3-cell coculture compared to culture alone. The expression of *CCL5* was significantly increased by 15.07-fold in THP-1 cells in the 3-cell coculture condition (Fig. 2B). Instead, *IL-6*, *IL-8* and *CCL5* levels were not increased in adipocytes i.e. OA, 3T3L1 adipocytes in 3-cell coculture condition (Fig. 2B). The similar results were observed in coculture with another OC cell line, OVCAR4 cell, and another U937 macrophages, as shown in Additional file 1: Fig. S2C-F. We demonstrated that in 3-cell coculture system, or omental TME of OC, ovarian cancer cells released both IL-6, IL-8, while CCL5 was from macrophages. We observed CCL5 induced modest pyroptosis effect i.e. less expression of cleavage form GSDMD, compared with IL-6 and IL-8 did (Additional file 1: Fig. S2G). Therefore, we next focus on OC-derived IL-6 and IL-8 effect in adipocyte pyroptosis.

We then measured the concentrations of IL-6 and IL-8 in 3-cell coculture CM with or without OC (Fig. 2C). The concentrations of IL-6 of OA/THP-1/SKOV3 CM, and 3T3L1/THP-1/SKOV3 CM were higher than that in OA/THP-1/iNEC CM and 3T3L1/THP-1/iNEC CM, which was 370.35 ± 20.65, 63.26 ± 0.69 vs. 167.29 ± 8.55, 0.88 ± 0.31 pg/ml, respectively. The concentrations of IL-8 of OA/THP-1/SKOV3 CM, and 3T3L1/THP-1/SKOV3 CM were also higher than that in OA/THP-1/iNEC CM and 3T3L1/THP-1/iNEC CM, which was 208.93 ± 3.77, 189.15 ± 3.71 vs. 177.96 ± 5.79, 141.48 ± 6.53 pg/ml, respectively (Fig. 2C).

For understanding the effects of IL-6, IL-8 in adipocytes, we treated OA and 3T3L1 adipocytes with recombinant IL-6 and IL-8, and checked the change of caspase-1 activity and NLRP3/caspase-1/GSDMD pathway in adipocytes (Fig. 2D, E). The caspase-1 activity of OA increased 20.78% after IL-6 (concentration: 370 pg/ml) treatment and 17.37% after IL-8 (concentration: 209 pg/ml) treatment, while increased 20.90% after combined IL-6 and IL-8 treatment (Fig. 2D, left panel). The caspase-1 activity of 3T3L1 adipocytes increased 25.48% after IL-6 (concentration: 63 pg/ml) treatment and 19.17% after IL-8 (concentration: 189 pg/ml) treatment, while increased 22.02% after combined IL-6 and IL-8 treatment (Fig. 2D, right panel). By Western blot analysis, the NLRP3/caspase-1/GSDMD was activated by increased cleavage -form of GSDMD in OA and 3T3L1 adipocytes after 3 day treatment of IL-6, IL-8 or combined IL-6 and IL-8 treatment (Fig. 2E).

For further understanding whether other GSDMs involved in the pyroptosis of this 3-cell culture system, we checked the change of GSDMB, GSDMC, GSDMD and GSDME expression level in SKOV3, THP-1 macrophages and adipocytes i.e. OA, 3T3L1 adipocytes after IL-6, IL-8 treatment. The results showed the cleavage form of GSDMD only increased in adipocyte under the IL-6 and IL-8 treatment, while the expression or cleavage form of GSDMB, GSDMC and GSDME did not change in SKOV3, THP-1 macrophages after IL-6 and IL-8 treatment (Additional file 1: Fig. S2H).

### Omental adipocyte releases ATP via pyroptosis contributes to macrophage infiltration

**Fig. 3:**
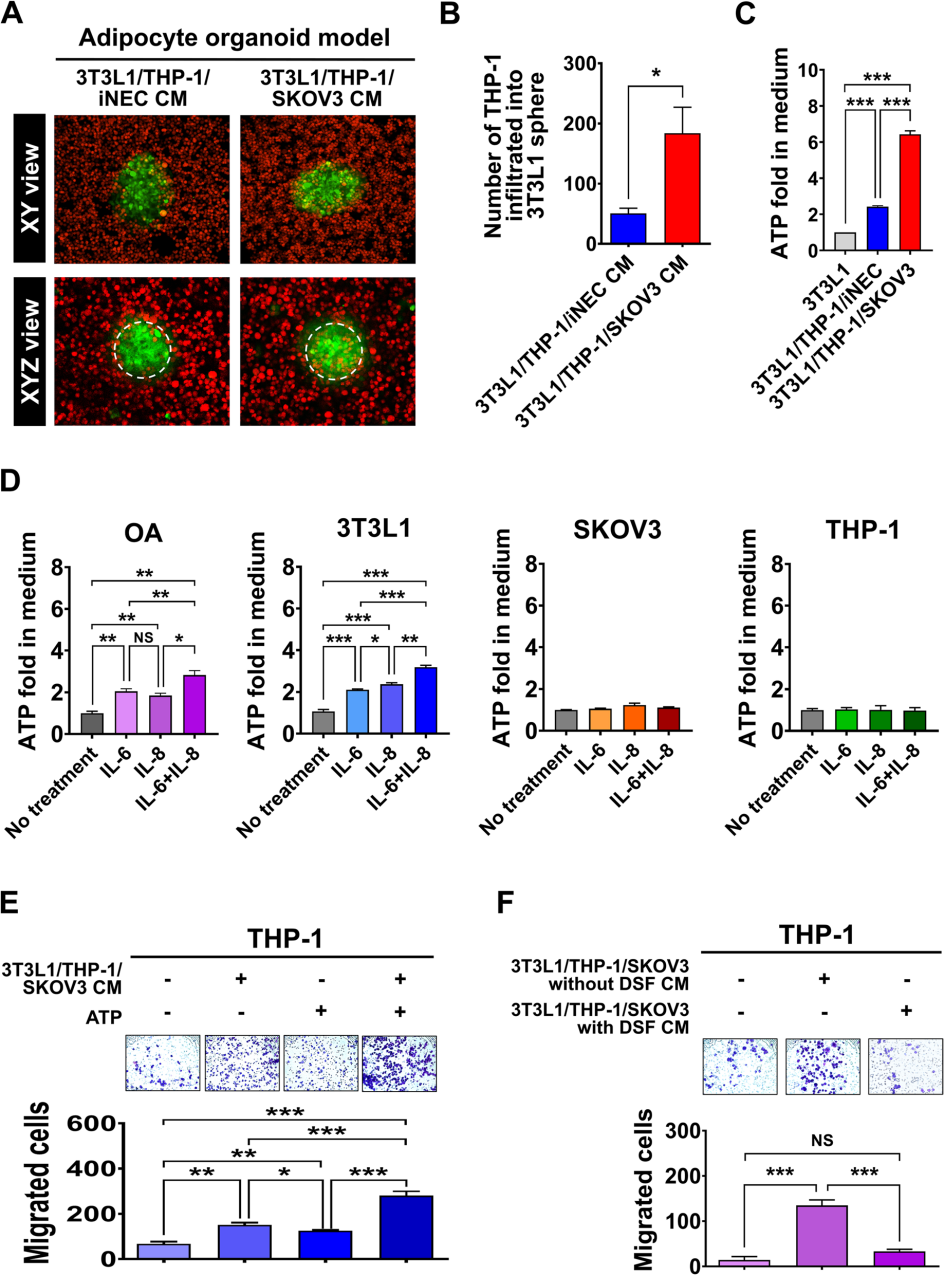
ATP released from adipocytes during pyroptosis induces macrophages migration to adipose tissue. **A**,** B** In the adipocyte organoid model, the number of infiltrated THP-1-RFP cells (red color) in 3T3L1-GFP spheres (green color) was significantly increased when treated with 3T3L1/THP-1/SKOV3 CM compared to treated with 3T3L1/THP-1/iNEC CM. Two-dimension view as XY view, three-dimension view as XYZ view which were cumulated from serial section of the adipocyte sphere at 1 μm at Z stack. The quantification of THP-1-RFP cells (red color) from XYZ view was showed in (**B**) with the mean values: 184 infiltrated macrophages when treated with 3T3L1/THP-1/iNEC CM vs. 51 infiltrated macrophages when treated with T3L1/THP-1/SKOV3 CM. **C** The ATP level in 3T3L1/THP-1/SKOV3 CM was significantly higher than in 3T3L1 adipocytes CM (1.00-fold) or in 3T3L1/THP-1/iNEC CM (2.4-fold) by luminescent ATP assay. **D** For determining the source of ATP in CM, OA, 3T3L1 adipocytes, THP-1 macrophages and SKOV3 cells were treated with IL-6, IL-8 or combination. After IL-6 or IL-8 or combination treatment, only adipocytes, i.e. OA or 3T3L1 adipocytes, the ATP level in CM were significantly increase. For SKOV3 or THP-1 macrophages, the ATP levels in CM were without change. **E** For macrophages cell migration assays, the number of migrated THP-1 macrophages cells was significantly increased after 3T3L1/THP-1/SKOV3 CM treatment (mean value: 150 cells), ATP (100 µM) treatment (mean value: 124 cells) and combination treatment (mean value: 280 cells), as compare with no treatment (mean value: 67 cells). **F** The mean number of migrated THP-1 macrophages cells was reduced from 134 to 33 after GSDMD inhibitor, DSF (0.3 µM) treatment. ∗*p* < 0.05; ∗∗*p* < 0.01; ∗∗∗*p* < 0.001

Previously, we have reported the number of omental CLS was associated with outcome of advanced-stage OC. To explore whether macrophage infiltration is influenced by adipocytes undergoing pyroptosis in the omental TME, we used an adipocyte organoid culture model to evaluate the changes in macrophage infiltration. 3T3L1-GFP spheres (Additional file 1: Fig. S3A) and THP-1-RFP cells were coculture with 3T3L1/THP-1/SKOV3 CM or 3T3L1/THP-1/iNEC CM to mimic the omental environment with or without tumor. After coculture for 24 h, the XYZ imaging were analyzed and the whole procedure was videotaped (Fig. 3A). The number of THP-1-RFP cells in the 3T3L-1-GFP spheres was higher when coculture with 3T3L1/THP-1/SKOV3 CM than that coculture with 3T3L1/THP-1/iNEC CM (mean ± SE: 183 ± 43 cells vs. 50 ± 8.5 cells; *p* = 0.03) (Fig. 3B) (Additional file 2: video 1_3T3L1 adipocytes sphere treated with 3T3L1/THP-1/iNEC CM, Additional file 3: video 2_3T3L1 adipocytes sphere treated with 3T3L1/THP-1/SKOV3 CM).

Because macrophages could be recruited by ATP that release from dead cells, and then perform the “find-me” and “clearance” program [6], we next detected the ATP levels in CM from different coculture conditions. We found that ATP level was significantly increased to 6.43-fold in 3T3L1/THP-1/SKOV3 CM or 2.42-fold in 3T3L1/THP-1/iNEC CM compared with 3T3L1 CM (Fig. 3C). The similar results were also observed in the 3-cell coculture CM of another ovarian cancer cells OVCAR4 and U937macrophages (Additional file 1: Fig. S3B). Next, we used recombinant IL-6 and IL-8 treat OA, 3T3L1 adipocytes, SKOV3 and THP-1 cells to find whether the ATP was from adipocyte or not. The results showed that the ATP levels in medium of adipocytes, OA, 3T3L1 adipocytes significantly increased after IL-6, IL-8 or combined Il-6, IL-8 treatment, while the ATP levels were not changed in the mediums of SKOV3, THP-1 macrophages (Fig. 3D).

For further understanding the effect of ATP for macrophages, we performed migration assay of THP-1 cells. Compared with no CM treatment, the migrated THP-1 cells significantly increase after the 3T3L1/THP-1/SKOV3 CM treatment (Mean ± SD, 67 ± 21 cells vs. 150 ± 24 cells, *p* = 0.0005), ATP (100 µM) treatment (124 ± 15 cells) and combined treatment (280 ± 42 cells) (Fig. 3E) (Additional file 1: Fig. S3C).

Since disulfiram (DSF) can inhibit GSDMD-dependent cell pyroptosis, we used DSF in the 3-cell coculture condition to reduce ATP level (Additional file 1: Fig. S3D) and to detect the change of macrophage migration. The results showed that the number of migrated THP-1 macrophages cells significantly decreased after CM from 3T3L1/THP-1/SKOV3 with DSF compared to that after CM from the 3T3L1/THP-1/SKOV3 without DSF (Mean ± SD: 32 ± 9 vs. 135 ± 21 migrated cells) (Fig. 3F). These results suggested that pyroptotic adipocyte-released ATP affects macrophages migration.

### FFA from pyroptotic adipocytes increases ovarian cancer cell invasion and chemoresistance

**Fig. 4:**
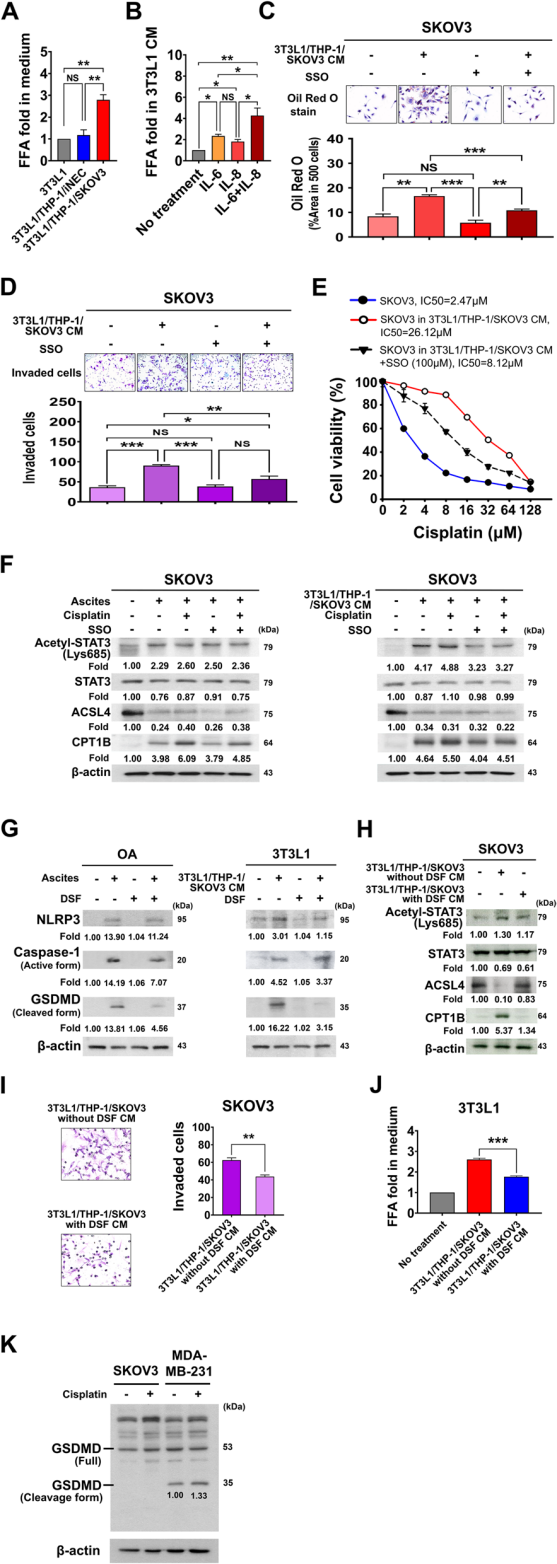
FFA released from adipocytes via pyroptosis contributes to invasion and chemoresistance of OC. **A** The FFA in 3T3L1/THP-1/SKOV3 CM was significantly elevated than that in 3T3L1 adipocytes CM or 3T3L1/THP-1/iNEC CM (2.79-fold vs. 1.00-fold vs. 1.18-fold). **B** The FFA in 3T3L1 adipocytes CM with IL-6, IL-8 co-treatment was significantly elevated than that with no treatment (4.27-fold vs. 1.0-fold), with IL-6 (2.33-fold) or IL-8 treatment (1.81-fold). **C** The lipid in SKOV3 cells significantly increased after 3T3L1/THP-1/SKOV3 CM treatment and decreased by FFA uptake inhibitor, SSO treatment. By Oil Red O staining, the area of positive stain in five hundred SKOV3 cells was significantly increased when treated with 3T3L1/THP-1/SKOV3 CM than those without CM (16.6% vs. 8.31%), while the area of positive stain decrease to 10.81% by SSO (100 µM ) treatment. **D** Increased SKOV3 FFA uptakes with 3T3L1/THP-1/SKOV3 CM, increases SKOV3 invasion ability. The mean number of invaded cells was significantly increased in SKOV3 after 3T3L1/THP-1/SKOV3 CM treatment as compared with control (90 cells vs. 36 cells), while the mean number of invaded cells was reduced to 57 cells when treated with SSO (100 µM). **E** Increased SKOV3 FFA uptakes with 3T3L1/THP-1/SKOV3 CM, increases SKOV3 cell chemoresistance. By MTT assays, the IC50 of cisplatin in SKOV3 cells was 2.47 µM but increased to 26.12 µM when treatment with 3T3L1/THP-1/SKOV3 CM. However, the IC50 of cisplatin in SKOV3 decreased to 8.12 µM when added with SSO (100 µM). **F** The chemoresistance of SKOV3 cultured in OC patient’s ascites or 3T3L1/THP-1/SKOV3 CM is associated involved fatty acid oxidation through elevated CPT1B expression. By Western blot analysis, the expressions of acetylated-STAT3, and CPT1B of SKOV3 cell treated with OC patient’s ascites or 3T3L1/THP-1/SKOV3 CM were increased, while reduced the expression after added with SSO (100 µM), i.e. CPT1B expression from 6.09-fold reduced to 4.85-fold in ascites cultured; 5.50-fold reduced to 4.51-fold in 3T3L1/THP-1/SKOV3 CM cultured. FFA released from adipocytes via pyroptosis contributes to invasion and chemoresistance of OC. **G** The DSF inhibits the NLRP3/caspase-1/GSDMD pathway of OA cultured in patient ascites and 3T3L1 adipocytes cells cultured in 3T3L1/THP-1/SKOV3 CM. The cleaved form of GSDMD was reduced from 13.81-fold to 4.56-fold in OA cells; from 16.22-fold to 3.15-fold in 3T3L1 cells by treated with DSF (0.3 µM). **H** DSF reversed fatty acid oxidation-related CPT1B expression of SKOV3 cell cultured in 3T3L1/THP-1/SKOV3 CM. The CPT1B expression was reduced from 5.37-fold to 1.34-fold by treated with DSF (0.3 µM). **I** The invaded SKOV3 cells significantly reduced by treated with DSF (0.3 µM) as compared without DSF treatment (mean invaded cells: 63 vs. 44 cells). **J** The FFA level in 3T3L1/THP-1/SKOV3 CM was significant decreased from 2.61-fold to 1.77-fold by treated with DSF (0.3 µM). **K** The SKOV3 cells did not occur pyroptosis by treated with cisplatin as no increase of cleaved form GSDMD. The breast cancer cell line, MDA-MB-231 as positive control for pyroptosis of cleaved form GSDMD

Adipocytes are well-known energy-storing cells and can release FFA into the extracellular environment as a result of cell death, stress or activation. We first checked the FFA level in CM with different coculture conditions. We found FFA level increase in 3T3L1/THP-1/SKOV3 CM with coculture duration, especially at Day 7 (Additional file 1: Fig. S4A). The FFA in 3T3L1/THP-1/SKOV3 CM was significantly elevated as compare with it in 3T3L1/THP-1/iNEC CM (2.79-fold vs. 1.18-fold when normalized with 3T3L1 adipocytes only CM, *p* = 0.0089) (Fig. 4A). Similar results were found when 3T3L1 adipocytes treated with recombinant IL-6 and IL-8. The FFA content in 3T3L1 CM was increase to 4.27-fold when treated with IL-6 and IL-8 compared to no IL-6, IL-8 treatment (*p* = 0.0098) (Fig. 4B). By Oil Red O staining, we found that FFA were can be taken up by SKOV3 cells after treatment with 3T3L1/THP-1/SKOV3 CM (Fig. 4C), and reversed by a CD36 inhibitor, sulfo-N-succinimidyl oleate (SSO) (100 µM) (Fig. 4C) (Additional file 1: Fig. S4B). The area of positive stain in five hundred SKOV3 cells was significantly decreased from 16.6 to 10.8% when treated with SSO (100 µM). We also observed the invaded cells of SKOV3 were significantly increased after 3T3L1/THP-1/SKOV3 CM treatment (Mean ± SD: 36 ± 7 vs. 90 ± 5 invaded cells; *p* < 0.0001), and reversed by SSO treatment (mean: 90 ± 5 vs. 57 ± 7 invaded cells; *p* = 0.005) (Fig. 4D).

Because suppressing fatty acid uptake with therapeutic effects has been reported in prostate cancer [19], we tested the therapeutic effects by suppressing fatty acid uptake in OC. By MTT assay, the IC50 of cisplatin in SKOV3 cells was 2.47 µM but increased to 26.12 µM when treatment with 3T3L1/THP-1/SKOV3 CM. However, the IC50 of cisplatin in SKOV3 decreased to 8.12 µM when added with SSO (100 µM) (Fig. 4E).

Recently, an important role of fatty acid oxidation in chemoresistance has been shown. Upregulated STAT3-induced carnitine palmitoyltransferase 1B (CPT1B) expression and fatty acid oxidation promoted breast cancer cell stemness and chemoresistance [20]. Acetylated STAT3 upregulated long-chain acyl-CoA synthetase 4 (ACSL4) and CPT1B to increase phospholipid biogenesis, elevate mitochondrial membrane potential and resisted chemotherapy-induced apoptosis [21]. Therefore, we studied whether fatty acid oxidation protecting cancer cells from apoptosis involved in our culture condition or not. We treated SKOV3 and OVCAR4 with OC patients’ ascites or 3T3L1/THP-1/SKOV3 CM and added with cisplatin, SSO or not. We observed the acetylated-STAT3/CPT1B were up-regulated and reversed by SSO (100 µM) (Fig. 4F) (Additional file 1: Fig. S4C).

To explore whether inhibition of adipocyte pyroptosis could reverse OC cells invasion in omental TME, we used pyroptosis inhibitor DSF in 3-cell coculture system. We found that both in OA and 3T3L1 adipocytes, the NLRP3/caspase-1/GSDMD pathway activation was suppressed by DSF (Fig. 4G). Furthermore, the expression of acetylated-STAT3 and CPT1B of SKOV3 cells were also reduced when treated with the 3T3L1/THP-1/SKOV3 CM with DSF (Fig. 4H). The SKOV3 cell invasion ability was significantly reduced in 3T3L1/THP-1/SKOV3 CM with DSF as compare with in 3T3L1/THP-1/SKOV3 CM without DSF (invaded cells, mean: 44 vs. 63 invaded cells, *p* = 0.0011) (Fig. 4I). From mediums without/with DSF, we found the FFA in medium significantly decreased from 2.61-fold to 1.77-fold (*p* < 0.0001) (Fig. 4J).

Cisplatin-induced tumor pyroptosis may contribute to anti-tumor effects in breast cancer [22]. Combination of DSF and cisplatin treatment may reduce the therapeutic effect of cisplatin if cisplatin-induced tumor pyroptosis also occurs in OC cells. Therefore, we studied whether cisplatin induce pyroptosis in OC cells or not. The results showed the cleavage form of GSDMD were not increased in SKOV3 and OVCAR4 cells after cisplatin (2 µM) treatment, while the cleavage form of GSDMD of breast cancer MDA-MB-231 cells increased to 1.33-fold after cisplatin treatment (Fig. 4K) (Additional file 1: Fig. S4).

### High pyroptosis in adipocytes of omental TME is associated with poor outcomes in advanced-stage OC

**Fig. 5:**
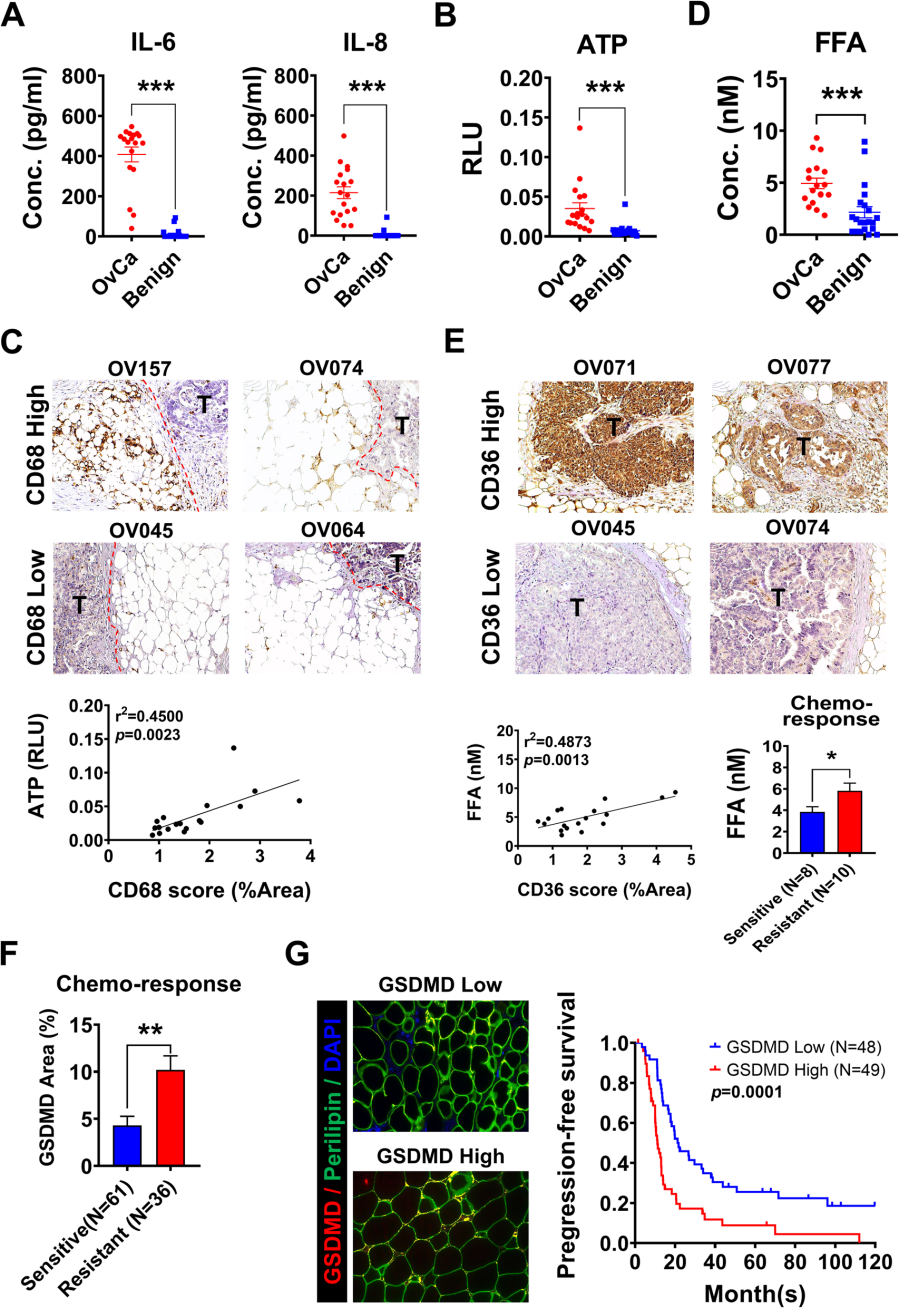
GSDMD-mediated pyroptosis in OC omental adipocyte causes chemoresistance and patient poor outcome. **A** The ascitic IL-6 (mean: 407.90 pg/ml) and IL-8 (mean: 214.80 pg/ml) in OC patients (*n* = 18) were higher than those in patients with benign disease (*n* = 21) (mean of IL-6 = 10.11 pg/ml; mean of IL-8 = 5.71 pg/ml). **B** The ascitic ATP level in OC patients (*n* = 18) (mean RLU = 0.0351) was significantly higher than that in patients with benign disease (*n* = 21) (mean RLU = 0.0071). **C** CD68 (+) macrophages in omental adipose tissue showed a positive correlation with the ascitic ATP level in OC patients (*n* = 18, *r*^2^ = 0.4500, *p* = 0.0023). **D**,** E** The level of ascitic FFA in OC patients (*n* = 18) (mean: 4.94 nM) was significantly higher than that in patients with benign disease (*n* = 21) (mean: 2.16 nM). CD36, as a receptor of FFA in OC cells, showed a positive correlation with the ascitic FFA concentration (*r*^2^ = 0.4873, *p* = 0.0013). Moreover, OC patients with high concentration of FFA in ascites was associated with chemoresistance (*p* = 0.0478) than those with low concentration of FFA in ascites. **F** In advanced-stage OC patients (*n* = 97), patient with chemoresistance (*n* = 36) had higher GSDMD expression in omental adipocytes than those with chemosensitive (*n* = 61) (10.19% vs. 4.29% area) (*p* = 0.0025). **G** For survival analysis, advanced-stage OC patient with high GSDMD expression in omental adipocytes carried a poor progression-free survival than those with low MSDMD expression (*p* = 0.0001)

Since concentrations of IL-6, IL-8 increased in our 3-cell coculture system and other reports from OC patients ascites [23, 24], we then checked the IL-6 and IL-8 levels in OC patients’ ascites by ELISA. Ascites from 18 advanced-stage OC patients and 21 patients with ovarian benign tumor were included. As compared with benign disease, the concentration of IL-6 and IL-8 were significantly increased in OC patient ascites, with mean value IL6: 407.88 vs. 10.11 pg/ml; IL8: 214.83 vs. 5.7 pg/ml (Fig. 5A). The ATP level was also significantly elevated in the ascites of OC patients compared with patients with benign disease (mean: 0.0351RLU vs. 0.0071RLU, *p* = 0.0003) (Fig. 5B). In addition, ATP levels in ascites were significantly positively correlated with the amount of omentum-infiltrating macrophages in the 18 OC patients (*r*^2^ = 0.4500, *p* = 0.0023) (Fig. 5C). The FFA concentration in the ascites of OC patients was significantly elevated compared with benign disease (mean value: 4.74 nM vs. 2.34 nM, *p* = 0.0007) (Fig. 5D) and positively correlated with CD36 expression in omental tumor (*r*^2^ = 0.4873, *p* = 0.0013) (Fig. 5E). More importantly, the OC patients with higher concentration of FFA in ascites were significantly associated with chemoresistance (*p* = 0.0478) (Fig. 5E, right lower panel). After excluding cases with omentum tissue broken during the process of staining, there were 97 cases preformed GSDMD staining of omentum and statistical analysis. The results showed that OC patients with high GSDMD expression in the omental adipocytes were associated with chemoresistance (*p* = 0.0025) (Fig. 5F) and poor progression-free survival (*p* = 0.0001) (Fig. 5G). These data indicates that pyroptosis occurs in OC omental adipocytes, and is positively correlated with levels of ATP and FFA in ascites. The OC patients with high omental GSDMD expression were associated chemoresistance and poor outcomes.

### Inhibition of adipocyte pyroptosis retards OC tumor growth, increases chemosensitivity and prolongs survival in vivo

**Fig. 6:**
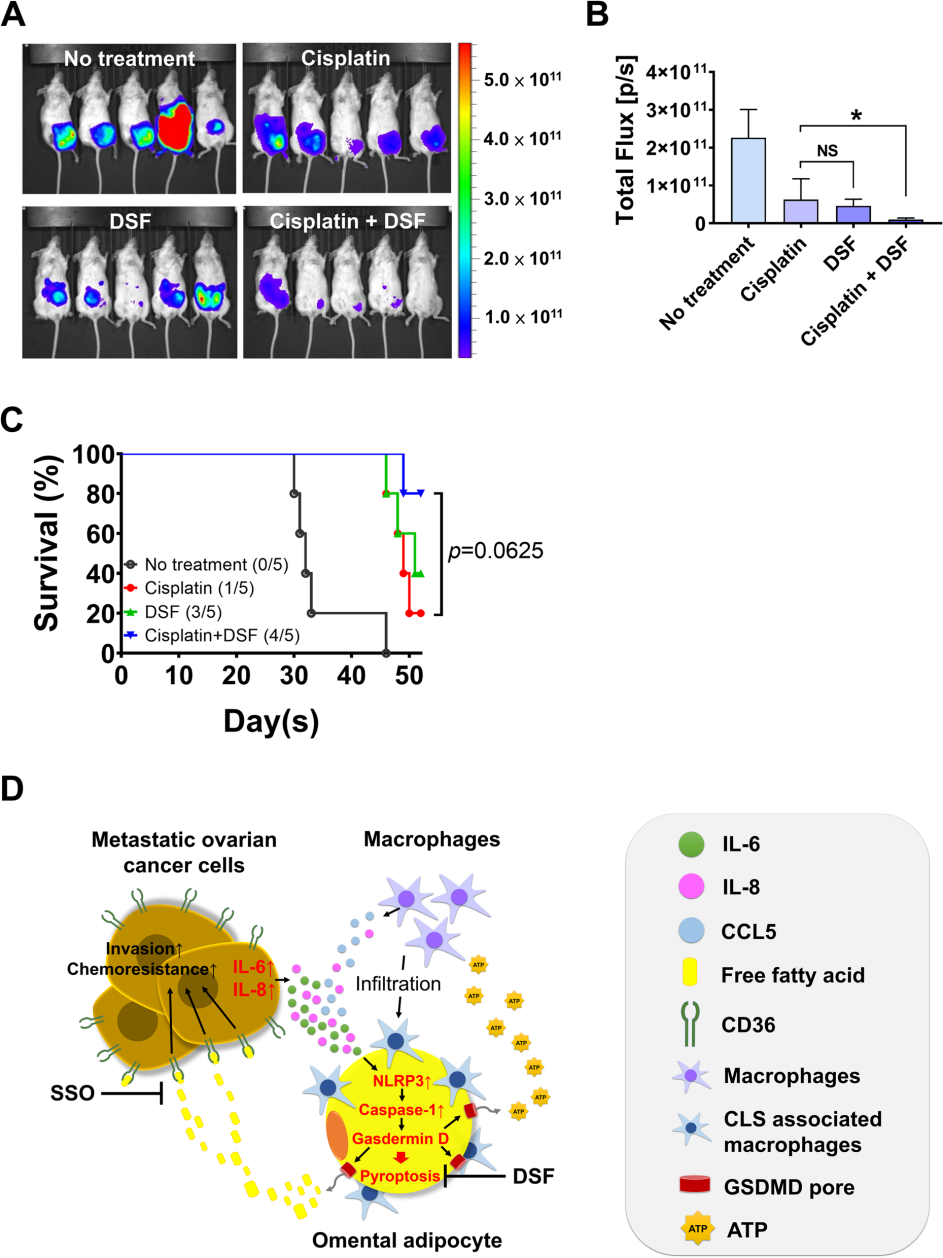
Inhibition of omental adipocyte pyroptosis is a potential therapeutic strategy in advanced-stage OC. **A**,** B** In the mouse model, tumor growth was retarded by cisplatin, DSF or combination treatment, especially DSF and cisplatin combination treatment. Quantification of the IVIS level at day 28 after tumor inoculation is shown in (**B**). **C** Survival analysis of the mice showed that inhibition of omental adipocyte pyroptosis prolonged survival in treated with DSF alone (survival rate = 60%) or combined with DSF and cisplatin treatment (survival rate = 80%). **D** Schematic representation of the proposed model for adipocyte pyroptosis in the omental TME of OC.

To evaluate the effect of pyroptosis inhibition of omental adipocyte in vivo, a xenograft mouse model was used. Mice were allocated into 4 groups including no treatment (group A), treated with cisplatin (4 mg/kg) (group B), treated with DSF (50 mg/kg) (group C), and treated with cisplatin and DSF (group D). Treatment started two weeks later after tumor inoculation. The results at day 28 showed that DSF treatment retarded tumor growth as effectiveness as cisplatin, while combined cisplatin and DSF treatment has best tumor control (Fig. 6A, B). For survival analysis in the 8th week after inoculation, the results showed a 60% survival rate in Group C (3/5 mice alive), and an 80% survival rate in Group D (4/5 mice alive) (Fig. 6C), which was better than Group A (0% survival rate, 0/5 mice alive) and Group B (20% survival rate, 1/5 mice alive). Results of immunofluorescence stain of GSDMD in mice omentum were showed on Additional file 1: Fig. S5. These data suggested that inhibition of omental adipocyte pyroptosis could control tumor growth, increased chemosensitivity and prolonged survival in mice model.

In summary, we demonstrated that in omental TME of OC, IL-6 and IL-8 released from tumor cells to trigger adipocyte pyroptosis and resulted in ATP released from omental adipocytes to promote macrophage infiltrate into omental TME; FFA released from omental adipocytes, uptake by tumor cells to increase cell invasion and chemoresistance. Inhibition of omental adipocyte pyroptosis may control OC tumor growth, increase chemosensitivity and improve survival. The graphic illustration of adipocyte pyroptosis in the omental TME of OC is shown in Fig. 6D.

## Discussion

In this study, we demonstrated that in omental TME, IL-6 and IL-8 from OC cells triggered adipocyte pyroptosis, and then released ATP and FFA to increase macrophage infiltration, cancer cell invasion and chemoresistance. Inhibition of omental adipocyte pyroptosis could control OC tumor growth, increase chemosensitivity and improve survival in the mice model. In OC, surgery and chemotherapy are currently the main therapeutic strategies but have only slightly improved the 5-year survival rates for advanced-stage disease over the past decades [25]. In contrast to melanoma, lung cancer, OC patients do not benefit from immunotherapy [26–28], which implies immune regulations in OC are still largely unknown. TME is a well-known complex milieu that varies in its cellular composition. In addition to 2-cell system [29–33], in this study, we try to establish an in vitro 3-cell coculture system including OC cells, adipocytes and macrophages to mimic the real omental TME in advanced disease of OC. We used immortalized normal uterine cervical epithelial cells (iNECs) as normal control cell. Although iNECs may not fully represent normal control cell for OC, there are some reasons we used them in this study. Morphologically, ovarian surface epithelium is a monolayered squamous-to-cuboidal epithelium [34] which may be partly similar with squamous epithelial tissues (cervix). In addition, some study used normal keratinocytes as control cells in ovarian cancer study [35].

By the 3-cell coculture system, we demonstrated that OC cell-derived IL-6 and IL-8 triggered adipocyte pyroptosis rather than apoptosis in omental TME. Secondly, we showed these ATP released from pyroptotic adipocytes play important role for macrophage infiltration into omental TME. Thirdly, we also observed FFA released from pyroptotic adipocytes is crucial for chemoresistance in OC which was through the acetylated-STAT3/CPT1B signal activation.

By qPCR, we clarify the IL-6 and IL-8 were released from OC cells rather than adipocytes in the 3-cell coculture system (Fig. 2B), although some reports suggested IL-6 and IL-8 could also release from inflamed adipocyte in obesity [36, 37]. Here, we used three different types of adipocytes, including one primary culture of human omentum (OA cell), one from differentiated mouse embryonic fibroblast cell (3T3L1 cell), and one differentiated human adipose-mesenchymal derived stem cells. Among these three cells, we did not observe the increase of transcription level of IL-6 and IL-8 after cocultured with THP-1/SKOV3 (Fig. 2B) (Additional file 1: Fig. S2F). From our cytokine array results in Fig. 2A, other cytokines were also produced by OC, adipocytes, macrophages, or 3-cell coculture. The IL-6 and IL-8 were secreted by ovarian cancer cells, while the CCL5 is mainly expressed in macrophages. The tumor-associated macrophages derived CCL5 has been reported to promote chemo-resistance and distant metastasis in prostatic cancer [38]. In addition, the CXCL5 (located on cytokine array E1 and E2), GRO (located on cytokine array H1 and H2), GRO-α (located cytokine array I1 and I2) and leptin (located on cytokine array J7 and J8) were obviously increased after coculture. CXCL5 has been demonstrated release from cancer-associated mesothelial cells to further promote the metastasis of OC [39]. GRO-a (CXCL1) interacted with CXCR2 to induce proliferation in epithelial ovarian cancer cells by transactivation of the epidermal growth factor receptor [40]. Gu et al. demonstrated high leptin level enhances the chemoresistance of ovarian cancer to treatment with platinum in combination with PTX/TXT to cause poor outcome [41]. The pyroptosis has demonstrated a form of inflammatory programed cell death pathway through caspase-1 activation and GSDMD cleavage to promote GSDMD pore formation on cell membrane. But to date, no reports indicate that cytokines can induce cell pyroptosis. The phenomenon of cytokine induced-pyroptosis that found in this study need more experiments to demonstrate.

In infection condition, pyroptosis has reported with an essential role in macrophages which clear the pathogens via gasdermin activation after pathogen infection [42]. However, the canonical pyroptotic pathway was not activated of macrophages in omental TME, as shown in Additional file 1: Fig. S2H. The recombinant IL-6 and IL-8 can trigger cleavage of GSDMD in OA, 3T3L1 adipocytes, but not in THP-1 macrophages. The results suggest macrophages of omental TME did not occur pyroptosis by IL-6 and IL-8. Notably, the combination of IL-6 and IL-8 could not achieve the same levels of caspases 1 and GSDMD activation as did the 3-cell co-culture CM in 3T3L1 (Fig. 2E). This indicated that other factors in the co-culture CM may facilitate the process, and need more experiments in the future.

Xu et al. reported that macrophage-derived IL-8 could promote colorectal cancer cell invasion via autocrine TNF-α regulation [43] and Lin et al. revealed tumor-associated macrophages-derived IL-8 was associated with poor outcome in gastric cancer via induction of PD-L1 + macrophages [44]. Indeed, the RNA levels of IL-8 in THP-1, U937macrophages were substantially increased when we used OVCAR4 cells in the coculture system (Additional file 1: Fig. S2B, C). Since ATP and FFA could release from 3T3L1 adipocytes after recombinant IL-8 treatment (Figs. 3D and 4B), we will explore the role of IL-8 from tumor associated macrophage in omental TME in OC in the future study.

We provide a new concept that omental adipocytes occur pyroptosis by IL-6 and IL-8 from tumor cells in advanced-stage OC. Metastatic OC cells could hijack the adipocytes of omental TME, trigger adipocyte pyroptosis to release ATP and FFA via its GSDMD-formed pores, result in macrophage infiltration and chemoresistance of OC. Inhibition of omental adipocyte pyroptosis could be a potential therapeutic modality to reverse chemoresistance and improve prognosis in advanced-stage OC.

## Conclusions

To explore the interactions between cells in CLS in omental TME that associated with poor prognosis in advanced-stage OC, we established an in vitro 3-cell coculture system. We demonstrated OC cells have the dominance in omental TME, they could hijack adipocytes and trigger the occurrence of GSDMD-mediated pyroptosis. The pyroptotic adipocytes could release ATP and FFA via the GSDMD pores to cause the macrophage infiltration and chemoresistance of OC. Importantly, inhibition of omental adipocyte pyroptosis have therapeutic potential to increase chemosensitivity in advanced-stage OC.

### Supplementary Information

Additional file 1.

Additional file 2.

Additional file 3.

## Data Availability

Data sharing is not applicable to this article as no datasets were generated or analyzed during the current study.

## References

[1] Marchetti C, De Felice F, Romito A, Iacobelli V, Maria Sassu C, Corrado G, Ricci C, Scambia G, Fagotti A (2021). Chemotherapy resistance in epithelial ovarian cancer: mechanisms and emerging treatments. Semin Cancer Biol.

[2] Tan DS, Agarwal R, Kaye SB (2006). Mechanisms of transcoelomic metastasis in ovarian cancer. Lancet Oncol.

[3] Nieman KM, Kenny HA, Penicka CV, Ladanyi A, Buell-Gutbrod R, Zillhardt MR, Romero IL, Carey MS, Mills GB, Hotamisligil GS (2011). Adipocytes promote ovarian cancer metastasis and provide energy for rapid tumor growth. Nat Med.

[4] Germain N, Dhayer M, Boileau M, Fovez Q, Kluza J, Marchetti P (2020). Lipid metabolism and resistance to Anticancer Treatment. Biology (Basel).

[5] Liang YL, Lin CN, Tsai HF, Wu PY, Lin SH, Hong TM, Hsu KF (2021). Omental Macrophagic Crown-like structures are Associated with Poor Prognosis in Advanced-Stage Serous Ovarian Cancer. Curr Oncol.

[6] Wang J, Kubes P (2016). A Reservoir of mature cavity macrophages that can rapidly invade visceral organs to affect tissue repair. Cell.

[7] Yu P, Zhang X, Liu N, Tang L, Peng C, Chen X (2021). Pyroptosis: mechanisms and diseases. Signal Transduct Target Ther.

[8] Rao Z, Zhu Y, Yang P, Chen Z, Xia Y, Qiao C, Liu W, Deng H, Li J, Ning P (2022). Pyroptosis in inflammatory diseases and cancer. Theranostics.

[9] Lu L, Zhang Y, Tan X, Merkher Y, Leonov S, Zhu L, Deng Y, Zhang H, Zhu D, Tan Y (2022). Emerging mechanisms of pyroptosis and its therapeutic strategy in cancer. Cell Death Discov.

[10] Wang Q, Imamura R, Motani K, Kushiyama H, Nagata S, Suda T (2013). Pyroptotic cells externalize eat-me and release find-me signals and are efficiently engulfed by macrophages. Int Immunol.

[11] Kong Q, Zhang Z (2023). Cancer-associated pyroptosis: a new license to kill tumor. Front Immunol.

[12] Faria SS, Costantini S, de Lima VCC, de Andrade VP, Rialland M, Cedric R, Budillon A, Magalhães KG (2021). NLRP3 inflammasome-mediated cytokine production and pyroptosis cell death in breast cancer. J Biomed Sci.

[13] Halbert CL, Demers GW, Galloway DA (1991). The E7 gene of human papillomavirus type 16 is sufficient for immortalization of human epithelial cells. J Virol.

[14] Chou CY, Shen MR, Wu SN (1995). Volume-sensitive chloride channels associated with human cervical carcinogenesis. Cancer Res.

[15] Hsu KF, Wu CL, Huang SC, Hsieh JL, Huang YS, Chen YF, Shen MR, Chung WJ, Chou CY, Shiau AL (2008). Conditionally replicating E1B-deleted adenovirus driven by the squamous cell carcinoma antigen 2 promoter for uterine cervical cancer therapy. Cancer Gene Ther.

[16] Picon-Ruiz M, Marchal JA, Slingerland JM (2020). Obtaining human breast adipose cells for breast Cancer cell co-culture studies. STAR Protoc.

[17] Rotter V, Nagaev I, Smith U (2003). Interleukin-6 (IL-6) induces insulin resistance in 3T3-L1 adipocytes and is, like IL-8 and tumor necrosis factor-alpha, overexpressed in human fat cells from insulin-resistant subjects. J Biol Chem.

[18] Meza-Perez S, Randall TD (2017). Immunological functions of the Omentum. Trends Immunol.

[19] Watt MJ, Clark AK, Selth LA, Haynes VR, Lister N, Rebello R, Porter LH, Niranjan B, Whitby ST, Lo J (2019). Suppressing fatty acid uptake has therapeutic effects in preclinical models of prostate cancer. Sci Transl Med.

[20] Wang T, Fahrmann JF, Lee H, Li YJ, Tripathi SC, Yue C, Zhang C, Lifshitz V, Song J, Yuan Y (2018). JAK/STAT3-Regulated Fatty Acid β-Oxidation Is Critical for Breast Cancer Stem Cell Self-Renewal and Chemoresistance. Cell Metab.

[21] Li YJ, Fahrmann JF, Aftabizadeh M, Zhao Q, Tripathi SC, Zhang C, Yuan Y, Ann D, Hanash S, Yu H (2022). Fatty acid oxidation protects cancer cells from apoptosis by increasing mitochondrial membrane lipids. Cell Rep.

[22] Yan H, Luo B, Wu X, Guan F, Yu X, Zhao L, Ke X, Wu J, Yuan J (2021). Cisplatin induces pyroptosis via activation of MEG3/NLRP3/caspase-1/GSDMD pathway in Triple-negative breast Cancer. Int J Biol Sci.

[23] Lane D, Matte I, Rancourt C, Piché A (2011). Prognostic significance of IL-6 and IL-8 ascites levels in ovarian cancer patients. BMC Cancer.

[24] Matte I, Lane D, Laplante C, Rancourt C, Piché A (2012). Profiling of cytokines in human epithelial ovarian cancer ascites. Am J Cancer Res.

[25] Ghisoni E, Imbimbo M, Zimmermann S, Valabrega G (2019). Ovarian Cancer immunotherapy: turning up the heat. Int J Mol Sci.

[26] Champiat S, Ileana E, Giaccone G, Besse B, Mountzios G, Eggermont A, Soria JC (2014). Incorporating immune-checkpoint inhibitors into systemic therapy of NSCLC. J Thorac Oncol.

[27] Alme AK, Karir BS, Faltas BM, Drake CG (2016). Blocking immune checkpoints in prostate, kidney, and urothelial cancer: an overview. Urol Oncol.

[28] Mantia-Smaldone GM, Corr B, Chu CS (2012). Immunotherapy in ovarian cancer. Hum Vaccin Immunother.

[29] Steitz AM, Steffes A, Finkernagel F, Unger A, Sommerfeld L, Jansen JM, Wagner U, Graumann J, Müller R, Reinartz S (2020). Tumor-associated macrophages promote ovarian cancer cell migration by secreting transforming growth factor beta induced (TGFBI) and tenascin C. Cell Death Dis.

[30] Hagemann T, Wilson J, Burke F, Kulbe H, Li NF, Plüddemann A, Charles K, Gordon S, Balkwill FR (2006). Ovarian cancer cells polarize macrophages toward a tumor-associated phenotype. J Immunol.

[31] Zhang L, Conejo-Garcia JR, Katsaros D, Gimotty PA, Massobrio M, Regnani G, Makrigiannakis A, Gray H, Schlienger K, Liebman MN (2003). Intratumoral T cells, recurrence, and survival in epithelial ovarian cancer. N Engl J Med.

[32] John B, Naczki C, Patel C, Ghoneum A, Qasem S, Salih Z, Said N (2019). Regulation of the bi-directional cross-talk between ovarian cancer cells and adipocytes by SPARC. Oncogene.

[33] Etzerodt A, Moulin M, Doktor TK, Delfini M, Mossadegh-Keller N, Bajenoff M, Sieweke MH, Moestru SK, Auphan-Anezin N, Lawrence T (2020). Tissue-resident macrophages in omentum promote metastatic spread of ovarian cancer. J Exp Med.

[34] Auersperg N, Wong AS, Choi KC, Kang SK, Leung PC (2001). Ovarian surface epithelium: biology, endocrinology, and pathology. Endocr Rev.

[35] Shalev N, Kendall M, Anil SM, Tiwari S, Peeri H, Kumar N, Belausov E, Vinayaka AC, Koltai H (2022). Phytocannabinoid compositions from Cannabis Act synergistically with PARP1 inhibitor against Ovarian Cancer cells in Vitro and affect the wnt signaling pathway. Molecules.

[36] Derosa G, Fogari E, D’Angelo A, Bianchi L, Bonaventura A, Romano D, Maffioli P (2013). Adipocytokine levels in obese and non-obese subjects: an observational study. Inflammation.

[37] Zagotta I, Dimova EY, Debatin KM, Wabitsch M, Kietzmann T, Fischer-Posovszky P (2015). Obesity and inflammation: reduced cytokine expression due to resveratrol in a human in vitro model of inflamed adipose tissue. Front Pharmacol.

[38] Ma J, Shayiti F, Ma J, Wei M, Hua T, Zhang R, Su J, Chen P (2021). Tumor-associated macrophage-derived CCL5 promotes chemotherapy resistance and metastasis in prostatic cancer. Cell Biol Int.

[39] Peng Y, Kajiyama H, Yuan H, Nakamura K, Yoshihara M, Yokoi A, Fujikake K, Yasui H, Yoshikawa N, Suzuki S (2019). PAI-1 secreted from metastatic ovarian cancer cells triggers the tumor-promoting role of the mesothelium in a feedback loop to accelerate peritoneal dissemination. Cancer Lett.

[40] Bolitho C, Hahn MA, Baxter RC, Marsh DJ (2010). The chemokine CXCL1 induces proliferation in epithelial ovarian cancer cells by transactivation of the epidermal growth factor receptor. Endocr Relat Cancer.

[41] Gu F, Zhang H, Yao L, Jiang S, Lu H, Xing X, Zhang C, Jiang P, Zhang R (2019). Leptin contributes to the taxol chemoresistance in epithelial ovarian cancer. Oncol Lett.

[42] Chen KW, Demarco B, Ramos S, Heilig R, Goris M, Grayczyk JP, Assenmacher CA, Radaelli E, Joannas LD, Henao-Mejia J (2021). RIPK1 activates distinct gasdermins in macrophages and neutrophils upon pathogen blockade of innate immune signaling. Proc Natl Acad Sci U S A.

[43] Xu H, Lai W, Zhang Y, Liu L, Luo X, Zeng Y, Wu H, Lan Q, Chu Z (2014). Tumor-associated macrophage-derived IL-6 and IL-8 enhance invasive activity of LoVo cells induced by PRL-3 in a KCNN4 channel-dependent manner. BMC Cancer.

[44] Lin C, He H, Liu H, Li R, Chen Y, Qi Y, Jiang Q, Chen L, Zhang P, Zhang H (2019). Tumour-associated macrophages-derived CXCL8 determines immune evasion through autonomous PD-L1 expression in gastric cancer. Gut.

